# Defoliation and fertilisation differentially moderate root trait effects on soil abiotic and biotic properties

**DOI:** 10.1111/1365-2745.14215

**Published:** 2023-10-25

**Authors:** Yan Liu, Irene Cordero, Richard D. Bardgett

**Affiliations:** ^1^ Department of Earth and Environmental Sciences The University of Manchester Manchester UK; ^2^ Department of Community Ecology Swiss Federal Institute for Forest, Snow and Landscape Research WSL Birmensdorf Switzerland

**Keywords:** growth strategy, perturbations, plant traits, plant–soil interactions, soil aggregates, soil microbial community, soil nutrients

## Abstract

1. Root functional traits are known to influence soil properties that underpin ecosystem functioning. Yet few studies have explored how root traits simultaneously influence physical, chemical, and biological properties of soil, or how these responses are modified by common grassland perturbations that shape roots, such as defoliation and fertilisation.
2. Here, we explored how root traits of a wide range of grassland plant species with contrasting resource acquisition strategies (i.e. conservative vs. exploitative strategy plant species) respond to defoliation and fertilisation individually and in combination, and examined cascading impacts on a range of soil abiotic and biotic properties that underpin ecosystem functioning.
3. We found that the amplitude of the response of root traits to defoliation and fertilisation varied among plant species, in most cases independently of plant resource acquisition strategies. However, the direction of the root trait responses (increase or decrease) to perturbations was consistent across all plant species, with defoliation and fertilisation exerting opposing effects on root traits. Specific root length increased relative to non‐perturbed control in response to defoliation, while root biomass, root mass density, and root length density decreased. Fertilisation induced the opposite responses. We also found that both defoliation and fertilisation individually enhanced the role of root traits in regulating soil biotic and abiotic properties, especially soil aggregate stability.
4. ***Synthesis***: Our results indicate that defoliation and fertilisation, two common grassland perturbations, have contrasting impacts on root traits of grassland plant species, with direct and indirect short‐term consequences for a wide range of soil abiotic and biotic properties that underpin ecosystem functioning.

## Graphical abstract

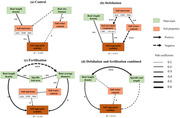

## 摘要

1. 众所周知，根系的功能性状会影响支撑生态系统功能的土壤性质。然而，很少有研究探讨各根系性状如何同时影响土壤的物理、化学和生物特性，以及这些反应如何被常见的草地扰动(例如落叶和施肥)所影响。
2. 这篇文章探讨了落叶和施肥如何单独和共同地影响具有相反资源获取策略(即保守性策略与获取性策略)的各种草原植物物种的根系性状，并研究了对支撑生态系统功能的非生物和生物土壤特性的一系列级联影响。
3. 我们发现根性状对落叶和施肥的反应幅度因植物种类而异，并在大多数情况下与植物资源获取策略无关。然而，所有研究的植物物种根性状对同一扰动的反应方向(增加或减少)都是一致的，并且落叶和施肥对根性性状具有相反的影响。相比于未扰动的对照组，落叶增加了比根长，但降低了根系生物量、根质量密度和根长密度。然而，施肥引起了相反的反应。我们还发现，落叶和施肥都单独地增强了根系性状在调节土壤生物和非生物特性中的作用，特别是土壤团聚体稳定性。
4. 总体上，我们的研究结果表明，落叶和施肥这两种常见的草原扰动对草原植物物种的根性状具有截然不同的影响，并对支撑生态系统功能的各种土壤非生物和生物特性产生直接和间接的短期影响。

## 1 INTRODUCTION

Plant roots play an irreplaceable role in shaping the soil environment via anchorage, penetration, and exudation of soluble, carbon‐rich compounds into soil (Bardgett et al., 2014; Freschet, Roumet, et al., 2021; Jin et al., 2017). Previous studies in grasslands show that root traits can impact a range of soil properties, including soil structure (Gould et al., 2016), nutrient availability (Dijkstra et al., 2021) and microbial community structure (Sweeney et al., 2021), although how these soil responses are modified by factors that can alter root traits, such as defoliation and fertilisation, remains unclear. This represents an important gap in knowledge given the prevalence of defoliation and fertilisation as grassland perturbations and their potential to influence root traits, with poorly understood consequences for the biotic and abiotic properties that underpin ecosystem functioning.

Both fertilisation and defoliation exert strong physiological effects on plants (Liu et al., 2018; Zheng et al., 2019). Defoliation removes photosynthetic shoot tissue and strongly influences plant biomass allocation patterns, root growth strategies, and root exudation, with consequences for a range of soil properties (Guitian & Bardgett, 2000; Hamilton & Frank, 2001; Medina‐Roldán & Bardgett, 2011). For example, defoliation of grassland plants is known to stimulate the short‐term release of root exudates, increasing soil microbial activity and nutrient availability (Hamilton & Frank, 2001; Mawdsley & Bardgett, 1997; Paterson & Sim, 2000). Also, microbial‐derived mucilages and root exudates can bond mineral particles together and form hydrophobic coatings on soil particles that will reduce slaking of soil aggregates (Czarnes et al., 2000; Hallett et al., 2009). Fertiliser application also changes nutrient availability to roots and can modify their growth strategies (Chen et al., 2020; Liu et al., 2019), and directly influences the soil microbial community (Leff et al., 2015; Ramirez et al., 2012). Moreover, fertilisation can stimulate fine root growth to allow plants to facilitate nutrient acquisition (Bordron et al., 2019; Wang et al., 2016). This enhancement of fine root production may improve soil aggregate stability by enmeshment and reduce macropore space via penetrating the soil (Bodner et al., 2014). Despite the known potential of defoliation and fertilisation individually to influence root traits, it remains unclear how they regulate root traits in combination and how root response to these perturbations cascade into soil chemical, biological and physical properties.

Root trait responses to perturbations and their consequences for soil properties are also likely to vary among plant species with different resource acquisition strategies. Grassland plant species vary considerably in both their root traits and their impact on soil properties and functions (De Long et al., 2019; de Vries & Bardgett, 2016; Grassein et al., 2015; Orwin et al., 2010). Species with exploitative resource acquisition strategies (characterised by fast growth rate and high specific leaf area) are associated with lower root dry matter content and root tissue density than conservative species (de Vries & Bardgett, 2016). This not only results in high nutrient demand of exploitative plants but also more vulnerability and less adaptive capacity to withstand perturbations. For example, faster‐growing, exploitative plants have high nutrient uptake capacities and rapid growth rates, and are typically associated with high rates of soil microbial activity, root exudation, soil organic nutrients mineralisation and nutrients desorption in their rhizosphere (Cui et al., 2019; Grigulis et al., 2013; Williams & de Vries, 2020). In particular, organic compounds such as carboxylate released by exploitative plants have been reported to influence nutrient availability in the rhizosphere (Faucon et al., 2017; Wen et al., 2022). Therefore, the growth of exploitative plant species might be hindered when perturbations are applied due to their high requirement for soil nutrients and water, and/or their low tolerance to stress (da Silveira Pontes et al., 2015; Pérez‐Ramos et al., 2017).

Plant species also differ in their capacity of nutrient uptake and in how they affect soil microbial communities and soil organic matter (SOM) stabilisation through their root activities and root‐mycorrhizal associations, which may result in variations in soil aggregate stability (Faucon et al., 2017; Hartmann & Six, 2023; Rillig et al., 2015; Wang et al., 2020). Soil nutrients are essential for the formation of soil aggregates, as they act as inorganic and organic binding agents that bond soil particles together, with potential impacts on aggregate stability (Ran et al., 2021). In addition, chemical binding action of microbial compounds, as well as physical enmeshment of fungal hyphae, could enhance soil aggregate stability (Merino‐Martín et al., 2021). Soil aggregation is fundamental in supporting soil functioning and ecosystem services, such as carbon sequestration, primary productivity and erosion prevention (Liu et al., 2021; Or et al., 2021; Rillig et al., 2015). Therefore, soil aggregate stability has been considered as a key parameter in assessing soil health and sustainability (Lehmann et al., 2017). An increasing number of studies have considered the contributions of abiotic and biotic factors such as root traits, SOM, and fungal communities to soil aggregation (Demenois et al., 2018; Liu et al., 2021; Zhang et al., 2019). However, it remains unclear how inter‐specific variation in root traits directly and indirectly affects soil aggregate stability, and how soil responses are modified by defoliation and fertilisation.

Here, we carried out a glasshouse study using a broad range of grassland plant species with different resource acquisition strategies to evaluate the short‐term responses of their root traits to perturbations common to grasslands, namely defoliation and fertilisation. Moreover, we investigated how these changes in root traits in turn affected a suite of biotic and abiotic soil properties that underpin ecosystem functioning. We aimed to disentangle the complex pathways by which root traits impact soil properties and clarify their importance for soil aggregate stability. We tested the hypotheses that: (1) the responses of root traits and soil properties to perturbations vary among plant species, and the relative effects of defoliation and fertilisation (alone or in combination) on the root traits of exploitative species are greater than for conservative species; and (2) changes in root traits triggered by different perturbations modify soil aggregate stability directly and indirectly via modification of soil biological and chemical properties.

## 2 MATERIALS AND METHODS

### 2.1 Experimental set‐up

Soil was collected from a mesotrophic grassland at Lancaster University's Field Station (54°1′ N, 2°46′ W). The soil was classified as a brown earth silt loam of the Brickfield 2 Association (3.30% C, 0.28% N, 7.25% SOM, pH 5.30) (Avis & Harrop, 1983; de Vries et al., 2018; de Vries & Bardgett, 2016; Kaisermann et al., 2017). After removing stones, macrofauna and visible plant residues, including roots, soil was passed through a 4 mm sieve and homogenised to ensure similar initial soil structure for all pots. Approximately 450 g of sieved soil was packed into 384 individual square 0.5 L pot (8 cm wide, 9 cm deep), with bulk density of 0.72 g cm^−3^. Water holding capacity (WHC) of sieved and homogenised soil was measured before seedling establishment.

Six grass and six herb species were selected to represent different resource acquisition strategies common to temperate mesotrophic grasslands (Table 1). They were classified into three categories according to their specific leaf area (SLA; from de Vries & Bardgett, 2016; Table 1): (1) conservative and slow‐growing grasses and herbs with low SLA; (2) grasses and herbs with intermediate SLA; and (3) exploitative and fast‐growing grasses and herbs with high SLA. Seedlings of all plant species were germinated in a controlled growth chamber (16/8 h and 20/16°C day/night) for 4 weeks and subsequently each individual seedling was transplanted into the centre of a pot. After seedling establishment, all pots were kept at 60% WHC by watering on a balance to a desired weight once every 2 days during the whole experiment. Nevertheless, soil water content still fluctuated due to differences in plant water demand and evaporation rate among pots and treatments, especially with fertilisation.

**TABLE 1:** Twelve grassland species used in this experiment, with specific leaf area functional group and category (de Vries & Bardgett, 2016).

Number	Species	SLA	Functional group	Category
1	*Deschampsia cespitosa* (Dc)	18.5	Grass	Conservative (1)
2	*Festuca rubra* (Fr)	17.7	Grass	Conservative (1)
3	*Leucanthemum vulgare* (Lv)	22.1	Herb	Conservative (1)
4	*Plantago lanceolata* (Pl)	22.4	Herb	Conservative (1)
5	*Cynosurus cristatus* (Cc)	26.4	Grass	Intermediate (2)
6	*Lolium perenne* (Lp)	26.4	Grass	Intermediate (2)
7	*Centaurea nigra* (Cn)	26.3	Herb	Intermediate (2)
8	*Ranunculus acris* (Ra)	23.8	Herb	Intermediate (2)
9	*Agrostis capillaris* (Ac)	30.8	Grass	Exploitative (3)
10	*Phleum pratense* (Pp)	30.6	Grass	Exploitative (3)
11	*Leontodon hispidus* (Lh)	28.8	Herb	Exploitative (3)
12	*Rumex acetosa* (Ruma)	28.1	Herb	Exploitative (3)

Eight weeks after seedling establishment, pots were randomly divided into four replicated blocks and then subjected to treatments after 2 weeks. Each block had 12 species × 4 treatments × 2 replicates = 96 pots, which yielded 384 pots in total. One replicate in each block was employed to measure root traits and the other one to measure soil properties, due to the destructive nature of the sampling for roots and soil measurements. The treatments were: non‐perturbed control, defoliation, fertilisation, and defoliation and fertilisation combined (hereafter referred to as the combined treatment). All treatments were applied once every 2 weeks, for 8 weeks, and the experiment was harvested 2 weeks after the treatments were completed. The defoliation treatment involved cutting and removal of shoot material at 4 cm above the soil surface, which is a widely recommend sward height for grazed agricultural grasslands (Medina‐Roldán & Bardgett, 2011). For fertilisation treatments, a slow‐release fertiliser (Osmocote Pro 3–4 month Controlled Release Plant Food granules 17:11:10 NPK) was ground and dissolved in Milli‐Q water. Subsequently, 10 mL NPK fertiliser solution was slowly added to topsoil of each pot with a syringe, avoiding being sprinkled on leaves. The total amount of fertiliser N added during the whole treatment period (4 times, once every 2 weeks) per pot (0.016 g N pot^−1^) was equivalent to 100 kg N ha^−1^, which is a typical amount added to agricultural mesotrophic grasslands (Ward et al., 2016).

### 2.2 Plant analyses

At final harvest, SLA for each plant was measured as described in Pérez‐Harguindeguy et al. (2016). Briefly, healthy, fully expanded leaves of each plant species were cut and wrapped in moist paper, and immediately placed in sealed plastic bags, which were stored overnight at 4°C. Leaves were then scanned, and the leaf area was measured by ImageJ 1.52a software. Leaves were oven‐dried at 60°C for 72 h. Leaf dry weight was used to calculate SLA as the ratio of leaf area to leaf dry mass. Subsequently, leaves were ground with a ball mill for analysing leaf nitrogen (N) and carbon (C) content using an elemental analyser Vario EL Cube (Elementar, Hanau, Germany). Cut plant material at each clipping date was weighed after being dried at 60°C for 72 h. A final harvest was performed 2 weeks after the final defoliation treatment was applied. At this time, all above‐ground plant material was harvested, oven‐dried at 60°C for 72 h, and weighed to measure total above‐ground biomass along with all clippings to assess total above‐ground plant biomass over the entire experimental period. All plant traits were measured at final harvest and did not include clipped material, except for measurements of above‐ground plant biomass, which was calculated as the sum of clipped and harvested plant biomass dry weight.

Roots were carefully separated from soil and stored in 10% ethanol at 4°C until analysis. The intact root system of each pot was scanned (Epson flatbed scanner) and architectural and morphological root traits were measured using the WinRhizo® root analysis software (Regent Instruments Inc., Canada). Specific root length was calculated by dividing the total root length by the root dry biomass (cm g^−1^), root tissue density by dividing the root volume by the weight of the dry biomass (g cm^−3^), root length density by dividing the total root length by the soil volume (cm cm^−3^), root mass density by dividing the root dry biomass by the soil volume (g dm^−3^), and root dry matter content by dividing the dry weight by fresh weight (unitless). Subsequently, roots were dried with tissue paper and weighed (fresh biomass). After being oven‐dried at 60°C for 72 h, root samples were weighed (dry biomass), ground, and analysed for root C and N content using an elemental analyser Vario EL Cube (Elementar, Hanau, Germany). Root mass fraction was calculated as the ratio of root dry mass to total plant accumulated dry biomass (Medina‐Roldán & Bardgett, 2011). Tables S1 and S2 show the variation in plant traits over plant species under non‐perturbed control conditions.

### 2.3 Soil analyses

At final harvest, all soil samples in pots were collected and sieved (5 mm mesh) to remove roots, homogenised and then stored at 4°C until further analysis. For each sample, 10 g sieved soil was weighed and oven‐dried at 105°C for 24 h to measure gravimetric soil water content, and a sub‐sample was ground for measurement of total C and N and SOM content.

Soil aggregate stability was determined using Le Bissonnais (1996) and ISO (2012) methods, which include three treatments: fast wetting (slaking), slow wetting (microcracking), and mechanical breakdown. Due to the susceptibility of soil aggregates >2 mm to root activities (Gould, 2014; Tisdall & Oades, 1982), 2–5 mm aggregates were kept for testing soil aggregate stability rather than 3–5 mm fraction. After being air‐dried and sieved, 2–5 mm aggregates were dried at 40°C for 24 h to achieve a constant matric potential. Subsequently, aggregates were subjected to the three treatments described in detail by Le Bissonnais (1996) and ISO (2012). After each treatment, the >0.05 mm fraction was oven‐dried at 40°C for 48 h and dry‐sieved by hand on a column of six sieves: 2, 1, 0.5, 0.2, 0.1 and 0.05 mm. The soil aggregate stability was calculated as mean weight diameter (MWD), which is the sum of the mass fraction of soil remaining on each sieve after sieving multiplied by the mean aperture of the adjacent mesh (Le Bissonnais, 1996):

$$\mathrm{MWD}=\sum_{i=1}^{n}X_{i}W_{i}$$

where *W*_*i*_ is the proportion of each aggregate size fraction, *X*_*i*_ is the mean aperture of the adjacent mesh. A higher MWD indicates a higher aggregate stability.

Soil organic matter was measured by the loss on ignition method (550°C, 6 h) (Ball, 1964) in a furnace (Carbolite Gero, Germany) and total soil C and N content were determined by using an elemental analyser Vario EL Cube (Elementar, Hanau, Germany). To measure dissolved organic C (DOC), 5 g of fresh soil was shaken in 35 mL of Milli‐Q water 10 min at 200 rpm, centrifuged for 30 min, and filtered through 0.45 μm syringe filters. To measure plant available ammonium and nitrate, 5 g of fresh soil was shaken in 25 mL of potassium chloride 1 h at 200 rpm, centrifuged for 5 min, and filtered through Whatman 42 paper filters. Filtered extracts were measured in an Auto Analyser AA3 (Seal Analytical, UK) for N measurements and in a 5000A TOC‐L analyser (Shimadzu, Japan) for C measurements. Microbial biomass C was determined by chloroform‐fumigation extraction method (Brookes et al., 1985). Briefly, 5 g of fresh soil was fumigated with alcohol‐free CHCl_3_ for 24 h at room temperature, extracted with 25 mL of 0.5 M K_2_SO_4_ by shaking for 30 min at 200 rpm, centrifuged for 5 min, and filtered through Whatman 42 paper filters. Another un‐fumigated 5 g of fresh soil was also extracted with K_2_SO_4_ as detailed above. C concentrations of K_2_SO_4_ extracts were determined using a Shimadzu 5000A TOC‐L analyser (Shimadzu, Japan). Microbial biomass C was calculated as the difference between fumigated and unfumigated samples and transformed using *k*_*EC*_ 0.35 (Sparling & West, 1988) as a conversion factor for C.

To characterise soil microbial communities, PLFA analysis was carried out using the method based on Buyer and Sasser (2012). Briefly, PLFAs were extracted from 1.0 g of freeze‐dried soil, and fatty acids were transesterified and dissolved in 75 μL of hexane. The extracted fatty acids were analysed on an Agilent 7890A Gas chromatograph. Specific PLFAs were selected to represent fungal and bacterial fatty acids. Bacterial PLFA biomass were represented by the fatty acids i‐15:0, a‐15:0, 15:0, i‐16:0, 17:0, i‐17:0, 17:0‐cyclo, 18:1ω7, and 19:0‐cyclo, and fungal fatty acids were represented by 18:2ω6 (Bardgett et al., 1996; Baxendale et al., 2014; de Vries et al., 2012). The ratio of fungal to bacterial PLFA biomass was calculated as the ratio of 18:2ω6: bacterial PLFAs (Bardgett et al., 1996). Variation in soil physicochemical and biological properties over plant species under non‐perturbed control is shown in Table S3.

### 2.4 Statistical analyses

All statistical analysis was carried out in R 4.0.3 statistical environment (R Core Team, 2020). To meet the assumptions of normality and homogeneity of variances, Box–Cox transformation were used for specific root length, MWD (including the three treatments: fast wetting, slow wetting, mechanical breakdown) and soil water content. All the remaining variables were log transformed. To examine the effects of plant species and treatments on soil properties and plant traits, mixed effect models were performed with three fixed factors (plant species, fertilisation, defoliation) and one random factor (block) by using R package “nlme” (Pinheiro et al., 2019). In addition, the relative effect (RE) of treatments were calculated as follows:

$$\mathrm{RE}=\frac{\text{Treatment}-\text{Control}}{\text{Control}}$$

Here, we used the value for treatments as individual observations, while the value for non‐perturbed control was the mean of the four non‐perturbed control replicates. Positive values of RE indicated an increase relative to non‐perturbed control as a result of perturbation treatments, negative values a decrease. A *t* test was performed to compare the significant differences between non‐perturbed control and treatments with the *compare means* function from the “ggpubr” package (Kassambara, 2020).

To further examine the influence of root traits on soil aggregation, linear mixed‐effects (LME) models covariate analysis was employed by the package “nlme” in R (Pinheiro et al., 2019), with root traits as covariates. Root trait variables were selected by variance inflation factors (where VIF <5) using R *corvif* function (Zuur et al., 2009). To explore the effects of root traits on soil aggregate stability, we used sequential type 1 sum of squares models to fit root traits first. The models were selected by comparing Akaike information criterion (AIC) values. *Coef* function were employed to extract model coefficients.

**FIGURE 1:**
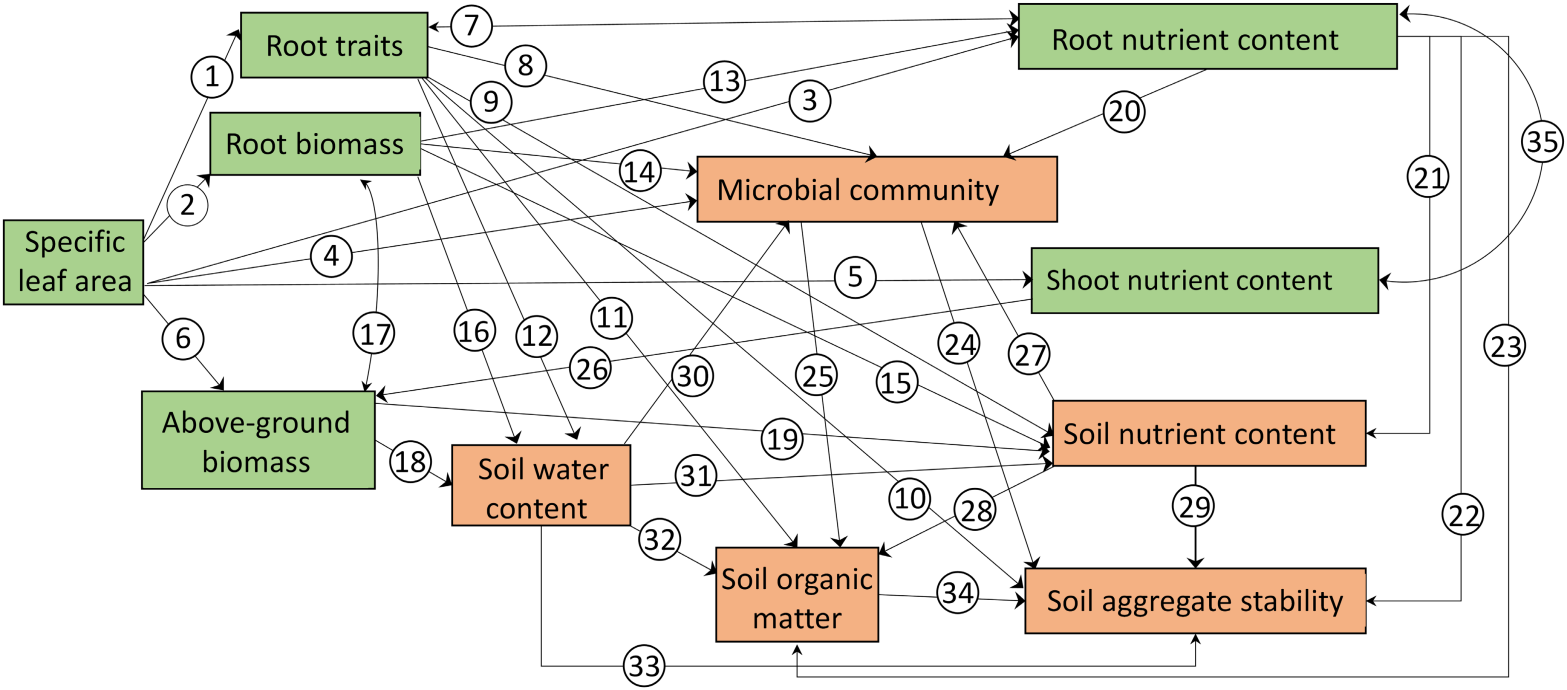
A priori model for effects of root traits and soil properties on soil aggregate stability. Green boxes represent plant properties and orange boxes represent soil properties. Specific leaf area (SLA), which reflects resource‐economic strategies of plant species, can affect root traits (arrow 1), root biomass (arrow 2), root nutrient content (arrow 3), microbial community (arrow 4), shoot nutrient content (arrow 5) and above‐ground biomass (AGB) (arrow 6) through species‐specific manner (Harrison & Bardgett, 2010; Herben et al., 2018; Metcalf et al., 2006). Root traits and root biomass can directly impact root nutrient content (arrow 7, arrow 13), microbial community (arrow 8, arrow 14), soil nutrient content (arrow 9, arrow 15), soil aggregate stability (mean weight diameter, MWD) (arrow 10), soil organic matter (arrow 11) and soil water content (SWC, arrow 12, arrow 16) through root activities, such as nutrient and water uptake, root exudation and anchorage (Bardgett et al., 2014; Jin et al., 2017). AGB can directly affect SWC (arrow 18) and soil nutrient content (arrow 19) through the uptake of water and nutrients from the soil (Zhang et al., 2020). Root nutrient content directly affects microbial community (arrow 20), soil nutrient content (arrow 21), MWD (arrow 22), SOM (arrow 23) and through root exudation (Hamilton et al., 2008). Soil microbial community can directly influence MWD through fungal hyphae (arrow 24) (Zhang et al., 2019), and it also affects the mineralisation of soil organic matter (SOM) (arrow 25) (Rousk et al., 2016). AGB accumulation can be affected by shoot nutrient content (arrow 26) (Yang et al., 2017). Soil nutrient content directly regulates microbial community activity (arrow 27) and the formation of SOM (arrow 28) and MWD (arrow 29) (Hunter et al., 2014; Ran et al., 2021). SWC regulates microbial activity (arrow 30) and soil nutrient content (arrow 31) (Cavagnaro, 2016; de Vries et al., 2018), and directly affects the formation of SOM (arrow 32) and soil aggregates (arrow 33) (Harrison‐Kirk et al., 2013; Xiao et al., 2018). SOM directly contributes to the increase of MWD (arrow 34) (Liu et al., 2021). Root nutrient content/root biomass is related to shoot nutrient content/AGB due to the nutrient competition between root and shoot (arrow 17, arrow 35) (Lamb et al., 2009).

To gain an integral understanding of the relative importance of leaf and root traits, soil chemical and biological properties for soil aggregate stability, structural equation modelling (SEM) was used. Our priori model was constructed to test our second hypothesis that root traits in responses to perturbations contribute to soil aggregate stability via affecting soil biological and chemical properties (Figure 1). Soil water content was included in the models (Figure 1) as a confounding factor directly and indirectly affecting soil aggregate stability. Plant biomass and root traits accounted for the variation in soil water content. To test the interlink between plant species and aggregate stability, SLA was used as a proxy for plant species in SEM models, given that plant species were selected according to their SLA. Soil aggregate stability was calculated as the average value of MWD under the three treatments (including fast wetting, slow wetting, and mechanical breakdown). Our models were fitted using the *sem* function from the “lavaan” package in R (Rosseel, 2012) and significant relationships with lowest AIC were kept when optimising models. The model fit was evaluated with Chi‐squared tests (*χ* ^2^, *p* > 0.05), comparative fit index (CFI > 0.95; Byrne, 1994), and root mean square error of approximation (RMSEA < 0.05; Pugesek et al., 2003; Steiger, 1989).

## 3 RESULTS

### 3.1 Effects of defoliation and fertilisation on plant traits

Most plant trait responses to defoliation and fertilisation (alone or in combination) were unrelated to plant resource acquisition strategies (Table S4). Root mass fraction of conservative species had the greater decrease than intermediate and exploitative species in responses to defoliation and fertilisation (alone). However, root average diameter of intermediate species had the greatest increase in responses to the combined treatment of defoliation and fertilisation. Leaf N content and leaf C: N ratio also varied with plant resource acquisition strategies in responses to fertilisation, with the greatest positive and negative responses of conservative species being detected, respectively.

**FIGURE 2:**
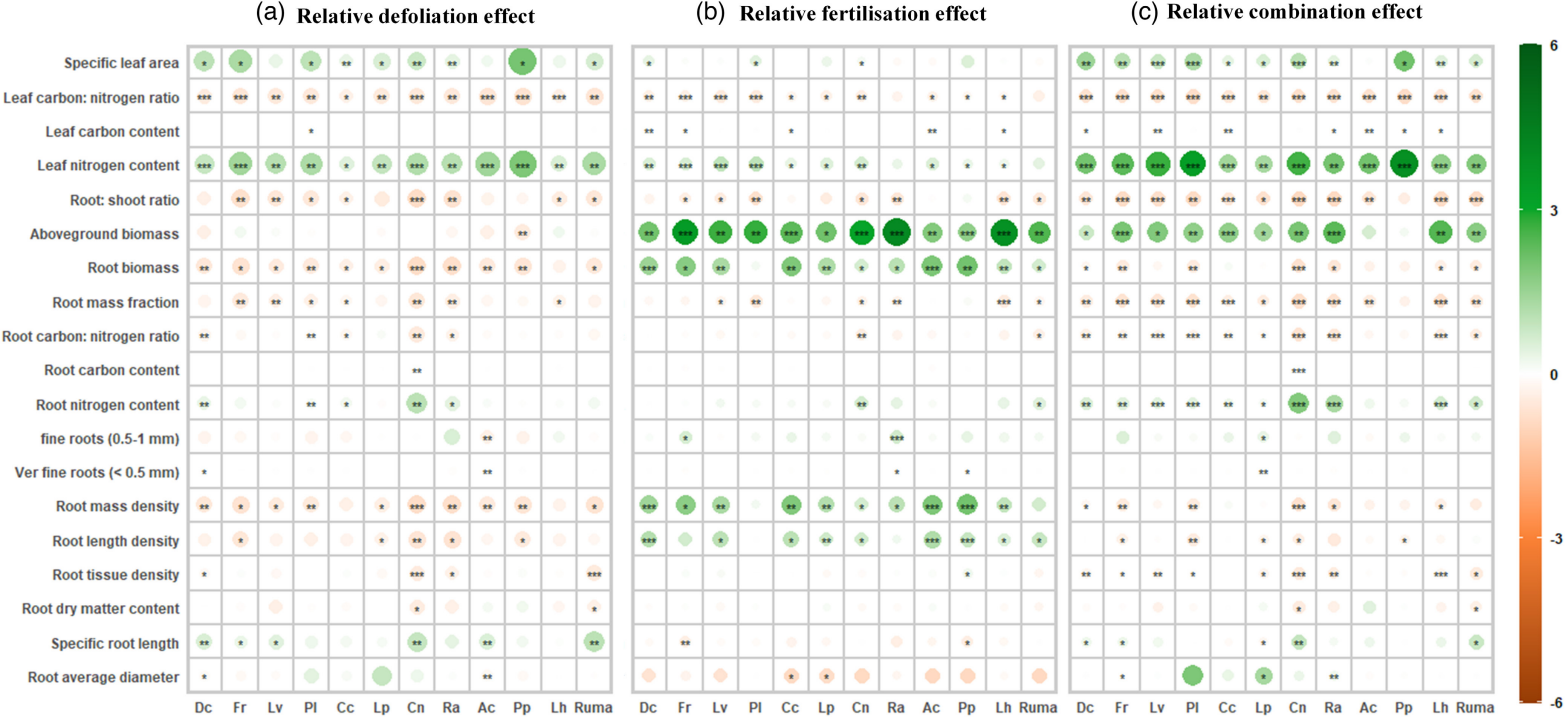
Relative perturbation effects on plant traits over 12 species (value = mean (*n* = 4)). (a) Relative defoliation effect; (b) relative fertilisation effect; (c) relative combination effect. Positive values indicate an increase relative to non‐perturbed control as a result of perturbation treatments, and negative values a decrease. The colour scale and associated values correspond to the direction and size of the perturbation effects. The size of the effect is also denoted by the circle size. Conservative species: *Deschampsia cespitosa* (Dc); *Festuca rubra* (Fr); *Leucanthemum vulgare* (Lv); *Plantago lanceolata* (Pl). Intermediate species: *Cynosurus cristatus* (Cc); *Lolium perenne* (Lp); *Centaurea nigra* (Cn); *Ranunculus acris* (Ra); Exploitative species: *Agrostis capillaris* (Ac); *Phleum pratense* (Pp); *Leontodon hispidus* (Lh); *Rumex acetosa* (Ruma). An asterisk indicates a significant difference between treatment and control. Levels of significance are: *p < 0.05, **p < 0.01, ***p < 0.001.

While the response of most leaf and root traits to defoliation varied significantly among species (Species × Defoliation, *p* < 0.05, Table 2), some consistent responses were detected. Across all plant species, specific root length increased, and root length density decreased in response to defoliation. Defoliation also decreased root mass density, root mass fraction, root biomass, root: shoot ratio and leaf C: N ratio, and increased leaf N content and SLA (Figure 2a). In contrast, fertilisation decreased specific root length and increased root length density across all species (Figure 2b). In addition, root mass density, root biomass, above‐ground biomass, and leaf N content were increased by fertilisation across all species (Figure 2b). While most effects of fertilisation on root traits were consistent across species, the amplitude of positive or negative effects on several plant traits varied among species (Species × Fertilisation, Table 2), such as average diameter, root mass density, root N content, leaf N content.

**TABLE 2:** Results of mixed effect models of the plant species, defoliation, fertilisation and all possible interactions on plant traits (block as random factor). *F* values and significant levels are given.

	Species	Defoliation	Fertilisation	Species × Defoliation	Species × Fertilisation	Defoliation × Fertilisation	Species × Defoliation × Fertilisation
AD	88.17***	33.99***	96.68***	3.43***	1.90*	0.17	0.94
SRL	85.25***	203.39***	60.42***	3.77***	2.56**	1.65	1.78
DMC	38.36***	32.04***	0.10	6.56***	1.15	0.76	0.61
RTD	92.56***	202.85***	8.77**	13.91***	0.91	11.01**	2.16*
RLD	70.61***	300.58***	115.49***	2.18*	1.69	12.96***	0.93
RMD	25.12***	745.07***	249.20***	6.02***	2.77**	23.38***	0.91
VFR	14.00***	3.00	46.00***	1.00	1.00	0.00	1.00
FR	14.64***	7.97**	46.64***	1.43	1.60	0.31	1.18
RNC	65.34***	131.70***	127.38***	7.27***	4.48***	3.61	0.77
RCC	12.00***	10.00**	3.00	1.00	1.00	18.00***	1.00
RCN	67.45***	123.18***	136.44***	7.19***	4.12***	1.63	0.83
RMF	34.76***	452.07***	274.36***	6.13***	8.18***	15.86***	0.69
AGB	31.89***	55.39***	1269.90***	3.38***	1.94*	19.46***	0.32
RB	29.47***	790.74***	255.17***	6.62***	3.34***	29.11***	0.81
RSR	36.62***	444.08***	260.78***	7.12***	6.50***	1.88	0.96
LNC	23.32***	1447.18***	487.96***	7.03***	3.41***	0.02	1.63
LCC	57.00***	1.00	75.00***	3.00**	2.00*	3.00	3.00**
LCN	26.44***	1444.49***	456.95***	6.77***	3.58***	0.00	1.61
SLA	20.37***	293.80***	13.67***	3.77***	1.55	0.84	1.19

Abbreviations: AD, root average diameter; AGB, above‐ground biomass; DMC, root dry matter content; FR, fine roots (0.5–1 mm); LCC, leaf carbon content; LCN, leaf carbon: nitrogen ratio; LNC, leaf nitrogen content; RB, root biomass; RCC, root carbon content; RCN, root carbon: nitrogen ratio; RLD, root length density; RMD, root mass density; RMF, root mass fraction; RNC, root nitrogen content; RSR, root: shoot ratio; RTD, root tissue density; SLA, specific leaf areaSRL, specific root length; VFR, very fine roots (<0.5 mm).

Asterisks show significance: *p < 0.05; **p < 0.01; ***p < 0.001.

Overall, the interactive effect of defoliation and fertilisation on plant traits did not vary among plant species (Defoliation × Fertilisation × Species, Table 2), and the direction of the effects was relatively consistent (Figure 2c). Additionally, we observed significant interactive effects of defoliation and fertilisation on several plant traits, such as root tissue density, root length density, above‐ground and root biomass (Defoliation × Fertilisation, Table 2). When defoliation and fertilisation were applied in combination, most plant traits responded in similar directions as under defoliation, except for above‐ground biomass, which increased as it did under fertilisation. The combined treatment increased leaf N content, with the amplitude of the increase being greater than that under defoliation or fertilisation alone.

### 3.2 Effects of defoliation and fertilisation on soil properties

**FIGURE 3:**
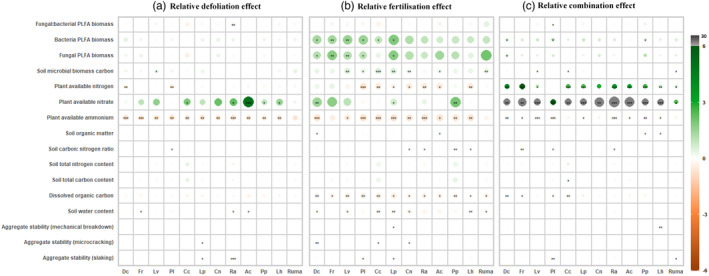
Relative perturbation effects on soil properties over 12 species (value = mean (*n* = 4)). (a) Relative defoliation effect; (b) relative fertilisation effect; (c) relative combination effect. Positive values indicate an increase relative to non‐perturbed control as a result of perturbation treatments, and negative values a decrease. The colour scale and associated values correspond to the direction and size of the perturbation effects. The size of the effect is also denoted by the circle size. Plant available nitrogen was calculated as the sum of plant available ammonium and nitrate. Conservative species: *Deschampsia cespitosa* (Dc); *Festuca rubra* (Fr); *Leucanthemum vulgare* (Lv); *Plantago lanceolata* (Pl). Intermediate species: *Cynosurus cristatus* (Cc); *Lolium perenne* (Lp); *Centaurea nigra* (Cn); *Ranunculus acris* (Ra); Exploitative species: *Agrostis capillaris* (Ac); *Phleum pratense* (Pp); *Leontodon hispidus* (Lh); *Rumex acetosa* (Ruma). An asterisk indicates a significant difference between treatment and control. Levels of significance are: *p < 0.05, **p < 0.01, ***p < 0.001.

The effects of defoliation on most soil properties were consistent among plant species, except for MWD1 (slaking) and plant available nitrate, which varied depending on plant species (Species × Defoliation, Table 3). Defoliation decreased MWD1 (slaking) of soil planted with *Ranunculus acris* and *Lolium perenne*, and greatly increased nitrate concentration in soil planted with *Agrostis capillaris* (Figure 3a). When considered independently for each plant species, defoliation only significantly decreased MWD2 (microcracking) in soil planted with *L. perenne* and significantly increased soil water content of the soil planted with *Festuca rubra*, *R. acris* and *A. capillaris* (Figure 3a), although an overall significant effect of these variables was detected (Table 3). Defoliation significantly decreased ammonium availability in soil planted with all plant species, except for *R. acetosa*, and significantly increased plant available nitrate in the soil planted with *Cynosurus cristatus*, *R. acris*, *A. capillaris*, *Phleum pratense* and *Leontodon hispidus* (Figure 3a). There was a weak trend, but significant overall (Table 3) of defoliation increasing soil microbial biomass C (Figure 3a) and decreasing the ratio of fungal to bacterial PLFA (Figure 3a).

**TABLE 3:** Results of mixed effect models of the plant species, defoliation, fertilisation, and all possible interactions on soil properties (block as random factor). *F* values and significant levels are given.

	Species	Defoliation	Fertilisation	Species × Defoliation	Species × Fertilisation	Defoliation × Fertilisation	Species × Defoliation × Fertilisation
MWD1	3.22**	0.15	1.45	2.35*	2.21*	6.61*	1.36
MWD2	0.54	46.98***	41.56***	1.29	0.44	31.26***	1.02
MWD3	0.96	27.43***	2.28	1.10	1.07	14.93***	1.00
SWC	2.23*	89.15***	74.17***	1.24	0.62	21.17***	1.35
DOC	1.63	2.03	93.88***	1.06	1.43	14.92***	0.41
TC	0.67	2.92	1.83	1.24	0.61	0.50	0.49
TN	0.81	1.61	0.03	1.31	0.62	0.32	0.56
CN	2.50**	5.60*	55.30***	1.00	0.90	0.50	1.00
SOM	1.77	1.82	33.22***	0.30	0.67	14.22***	0.51
NH_4_	0.79	203.38***	21.10***	0.77	1.56	332.99***	1.82
NO_3_	23.49***	402.92***	199.26***	4.65***	1.70	92.64***	0.72
MBC	1.66	6.04*	28.58***	1.14	0.43	54.29***	0.55
F	4.01***	8.10**	66.25***	0.68	0.38	5.90*	0.71
B	0.56	0.77	78.58***	0.39	1.01	14.96***	0.25
F/B ratio	13.14***	12.5**	0.48	0.72	1.19	2.65	0.88

Abbreviations: B, bacterial PLFA biomass; DOC, dissolved organic carbon; F, fungal PLFA biomass; F/B ratio, the ratio of fungal to bacterial PLFA biomass; MBC, soil microbial biomass carbon; MWD1, mean weight diameter (slaking); MWD2, mean weight diameter (microcracking); MWD3, mean weight diameter (mechanical breakdown); NH_4_, plant available ammonia; NO_3_, plant available nitrate; SOM, soil organic matter; SWC, gravimetric soil water content; TC, total soil carbon content; TN, total soil nitrogen content.

Asterisks show significance: *p < 0.05; **p < 0.01; ***p < 0.001.

Fertilisation significantly affected most soil variables, and these effects were consistent among species (Species × Fertilisation, Table 3), except for MWD1 (slaking). Fertilisation only decreased MWD1 (slaking) of soil planted with the herb *Plantago lanceolata* and the grass *L. perenne*. There was a trend of fertilisation decreasing soil water content, DOC and plant available ammonium across all species, although there was much inter‐specific variability in the amplitude of these responses (Figure 3b). In contrast to defoliation, fertilisation increased microbial biomass C, fungal PLFA biomass and bacterial PLFA biomass (Figure 3b).

There was an interactive effect of defoliation and fertilisation (Defoliation × Fertilisation, Table 3) on soil aggregate stability, including MWD (slaking) (*F* = 6.61, *p* < 0.05), MWD (microcracking) (*F* = 31.26, *p* < 0.001), MWD (mechanical breakdown) (*F* = 14.93, *p* < 0.001). The interactive effect of defoliation and fertilisation was also found on other soil properties, including soil water content, soil nutrients (DOC, SOM, plant available ammonium and nitrate), and microbial community (biomass C, fungal and bacterial PLFA). When defoliation and fertilisation were combined, there was a trend of decreasing soil concentrations of DOC and ammonium, and increasing soil nitrate, fungal PLFA, bacterial PLFA across all species (Figure 3c).

### 3.3 Determinants of soil aggregate stability

**FIGURE 4:**
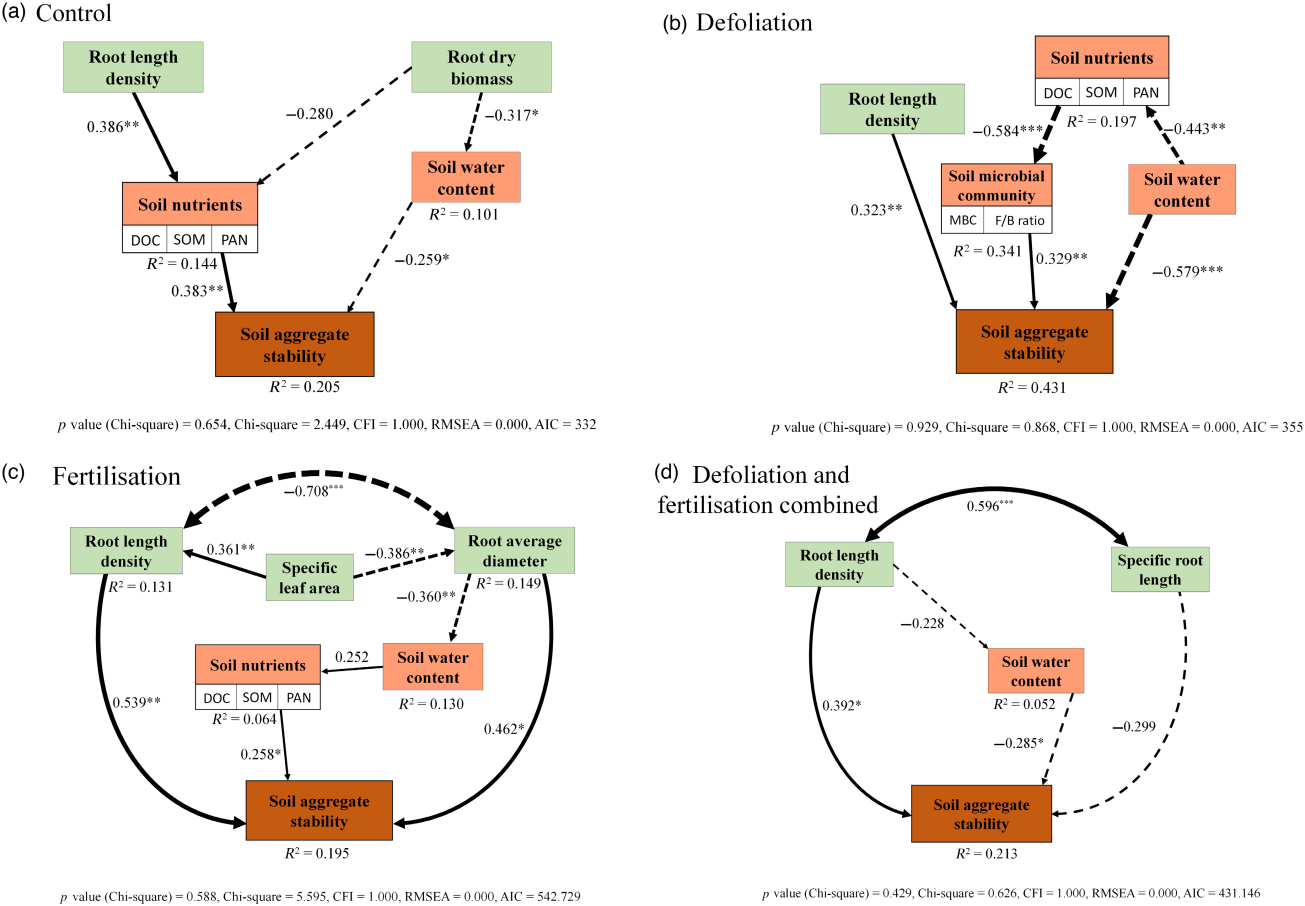
Structural equation model (SEM) showing the effects of root traits and soil chemical and biological properties on soil aggregate stability (the average of MWD under slaking, microcracking, and mechanical breakdown treatments). To gain an integral understanding of how perturbations affect the contributions of both root traits and soil properties to soil aggregate stability, this figure includes different treatments: (a) control; (b) defoliation, (c) fertilisation, (d) defoliation and fertilisation combined. Multiple‐layer rectangles represent the first component from the principal component analysis conducted for soil nutrients and soil microbial community. The variables underneath are the soil properties affecting mean weight diameter most and are selected for principal component analysis. Solid arrows represent positive relationships and dashed arrows represent negative relationships. Arrow width is proportional to a path coefficient of the relationship. DOC, dissolved organic carbon; SOM, soil organic matter; PAN, plant available nitrogen (ammonium + nitrate); MBC, microbial biomass carbon; F/B ratio, the ratio of fungal to bacterial PLFA biomass; CFI, comparative fit index; RMSEA, root mean square error of approximation. Levels of significance are: *p < 0.05, **p < 0.01, ***p < 0.001.

Both LME covariate analysis and SEM results revealed the importance of defoliation and fertilisation treatments in modifying the relative contributions of both root traits and soil properties to soil aggregate stability (Table 4; Figure 4). Increasing root length density was associated with increased MWD of both slaking and microcracking, while increasing specific root length was associated with decreased MWD of microcracking and mechanical breakdown. Increasing root average diameter was associated with decreased MWD of microcracking. After including root traits as covariates, the interactive effect of defoliation and fertilisation significantly affected MWD1 (slaking), MWD2 (microcracking), MWD3 (mechanical breakdown) (Table 4). The effects of defoliation and fertilisation on MWD1 (slaking) were dependent on plant species (Species × Defoliation, *F* = 2.11, *p* < 0.05; Species × Fertilisation, *F* = 1.98, *p* < 0.05), with the strong main effect of species on this measure (*F* = 2.82, *p* < 0.01).

**TABLE 4:** Results of linear mixed‐effects (LME) covariate analysis of the effects of root traits and perturbation treatments on soil aggregate stability (block as random factor). *F* values and significant levels are given. Root trait variables were selected by variance inflation factors (where VIF <5), including root average diameter, specific root length, and root length density.

	VIF	df	Soil aggregate stability (MWD, mm)		
			Slaking	Microcracking	Mechanical breakdown
*Covariates*					
AD	3.15	1	0.20	8.90**↓	1.05
SRL	2.83	1	0.03	14.00***↓	8.00**↓
RLD	1.21	1	6.86**↑	42.09***↑	1.77
*Factors*					
Species		11	2.82**	1.23	0.95
Defoliation		1	1.51	1.36	20.47***
Fertilisation		1	4.42*	14.21***	0.50
Species × Defoliation		11	2.11*	1.26	1.05
Species × Fertilisation		11	1.98*	0.47	0.99
Defoliation × Fertilisation		1	10.09**	32.49***	17.31***
Species × Defoliation × Fertilisation		11	1.20	0.89	0.82

*Note*: Text in bold means a significant effect and upper/lower arrows show covariates or factors corelated positively/negatively with soil aggregate stability. VIF shows the variance inflation factor of covariates.

Abbreviations: AD, root average diameter; SRL, specific root length; RLD, root length density.

Asterisks show significance: *p < 0.05; **p < 0.01; ***p < 0.001.

Increasing root length density was shown to be related to higher soil aggregate stability in the well‐fitted SEM models (Figure 4), which was also detected in the covariate analysis. Higher soil water content was directly associated with decreasing aggregate stability (Figure 4a,b,d), while higher soil nutrients were directly related to higher aggregate stability (Figure 4a,c). Soil microbial community only increased aggregate stability in defoliation model (Figure 4b).

The combination of root traits and soil nutrients explained 21%, 43%, 20%, 21% of the variance in soil aggregate stability in control, defoliation, fertilisation, and combined treatment models, respectively (Figure 4). Although soil properties directly or indirectly contributed to aggregate stability, the negative contributions of soil water content counteracted the positive effects of soil microbial community and soil nutrients (Figure 4). For example, in defoliation model (Figure 4b), the positive direct effect of soil microbial community on soil aggregate stability was 0.329 while the negative direct effect of soil water content was −0.579, resulting in the lower total direct effect of soil properties (0.25) than root traits (root length density, 0.323). Furthermore, in the fertilisation model (Figure 4c), the direct contributions of root traits to soil aggregate stability were higher than soil properties while the direct effects of soil properties in control model were higher than root traits.

## 4 DISCUSSION

Our overall goal was to experimentally test, across a broad range of grassland plant species with contrasting resource acquisition strategies, how root traits respond to defoliation and fertilisation individually and in combination, and to examine short‐term consequences for a wide range of soil properties that underpin ecosystem functioning. We found that the amplitude of the response of root traits to defoliation and fertilisation varied among plant species, in most cases independently of plant resource acquisition strategy. However, the direction of the root trait responses (increase or decrease) to perturbations was consistent across all plant species, with defoliation and fertilisation exerting opposing effects on root traits. We also found that the response of a range of biological, chemical and physical soil properties to defoliation and fertilisation was consistent among plant species. In addition, defoliation and fertilisation strongly shaped the contribution of root traits and soil biological and chemical properties to soil aggregate stability, which supported our second hypothesis. Together, our findings indicate that perturbations common to managed grasslands change root traits and their short‐term impacts on soil properties underpinning ecosystem functioning, including soil aggregate stability, a vital structural property of soil.

We found that root trait responses to perturbations were not related to plant resource acquisition strategy, which did not support our first hypothesis. We hypothesised that root trait responses of exploitative species to perturbations would be greater than conservative species. The inconsistency of our findings with our hypothesis is likely attributed to the complexity of root trait responses to perturbations. Indeed, a growing number of studies suggest that root trait variation is two‐dimensional or multidimensional and cannot be simply explained by one‐dimensional leaf economic spectrum from conservative to acquisitive strategies (Bergmann et al., 2020; Erktan et al., 2018; Zhou et al., 2018). For example, Kramer‐Walter et al. (2016) found that responses of root tissue density to soil fertility aligned with above‐ground traits such as SLA, but specific root length and root diameter were orthogonal to plant economic spectrum. Freschet et al. (2018) also found substantial heterogeneity in species plasticity and multiple facets of a wide range of above‐ and below‐ground responses to resource stress. Furthermore, the plant economic spectrum is often defined by leaf strategies that explain the ability of above‐ground plant organs to acquire resources by photosynthesis, rather than by below‐ground root strategies (Bergmann et al., 2020). However, plant responses to perturbations are substantially dependent on the ability of root systems to acquire soil water and nutrients or their interactions with mycorrhizal fungal partners after perturbations (Bennett & Groten, 2022; Bergmann et al., 2020; Freschet, Pagès, et al., 2021). Moreover, the complex root trait responses to perturbations might be attributed to multiple evolutionary processes and symbiotic partnerships of roots with arbuscular mycorrhizal fungi (Bergmann et al., 2020; Sobral, 2021).

### 4.1 Effects of perturbations on root traits

One important finding of our study was that relative to non‐perturbed control, specific root length increased in response to defoliation, while root biomass, root mass density, and root length density decreased. In contrast, fertilisation induced the opposite responses. Although the amplitude of defoliation and fertilisation effects on root traits varied among species, the detected responses demonstrate the potential of these perturbations to shape root traits in grassland systems, albeit in contrasting ways (Freschet et al., 2015; Fujita et al., 2010; Ma et al., 2020).

The consistent positive response of specific root length to defoliation observed across all plant species tested contrasts with previous studies reporting little or no response at all, albeit in a handful of plant species (Leuschner et al., 2013; Thorne & Frank, 2009). This inconsistency is likely attributed to differences in species‐specific survival strategies (Lepš et al., 2018; Qu et al., 2020). In our study, defoliation did not increase percentage of very fine roots (<0.5 mm) or fine roots (0.5–1 mm), which contrasts the notion that defoliated plants tend to invest more fine roots to increase nutrient capture (Eissenstat, 1992; Gordon & Jackson, 2000; Zadworny et al., 2016).

The opposite effect of fertilisation on root traits, such as decreased specific root length, may relate to nutrient acquisition strategies of plants, as well as high nutrient availability that could be invested into new root growth (Leuschner et al., 2013; Lugli et al., 2021; Ostonen et al., 2007). Previous studies have reported that fertilisation or high soil fertility shifts root traits from acquisitive to more conservative traits, such as lower specific root length but greater root average diameter and root tissue density (Bardgett et al., 2014; Lugli et al., 2021; Roumet et al., 2006), which was partially supported in our study. We found that fertilisation decreased specific root length, but also decreased root average diameter, and increased root mass density and root length density rather than root tissue density.

### 4.2 Effects of perturbations on soil properties

Our results indicate that defoliation and fertilisation individually and in combination, increased nitrate and decreased ammonium concentration in soil, which is in line with previous studies (Capstaff et al., 2021; Klaus et al., 2018; Wang et al., 2022). This likely relates to the role of the soil microbial community in the processes of nitrification and denitrification (Qin et al., 2019). For example, an increase in functional gene abundance of ammonium oxidising bacteria in response to fertilisation could accelerate transformation of ammonium into nitrate, resulting in lower pH, a decrease in ammonium, and an increase in nitrate in soil (Cameron et al., 2013; Yang et al., 2020; Zou et al., 2022). However, other studies have reported negative or neutral effects of defoliation on soil nitrate concentrations (Cameron et al., 2013; Louahlia et al., 2000). This inconsistency may relate to the difference of defoliation severity and plant age (Conrad‐Rooney et al., 2020; Wang et al., 2018). When defoliation and fertilisation were combined, soil nitrate concentration increased greatly, while ammonium concentrations decreased slightly, which is likely because N supply exceeded the demand of N by soil microbial community and plant growth.

Defoliation slightly but significantly increased microbial biomass C, but had little impact on DOC, which may relate to an increase in root exudation of defoliated plants (Hamilton et al., 2008). Although not directly measured, it is possible that organic compounds released in exudates of defoliated plants will have been utilised by the microbial community for biomass production, thereby increasing microbial biomass in the rhizosphere (Hamilton et al., 2008; Shen et al., 2020). Fertilisation decreased soil concentration of DOC, but increased soil microbial biomass, which is likely explained by an increase in nutrients availability for microbes, resulting in an increase of microbial C demand and a decrease of DOC in soil (Dai et al., 2018; Woolf & Lehmann, 2019). When combined, defoliation and fertilisation had a small, but significant impact on soil microbial biomass C and DOC. This response might be attributed to the changes in microbial activity and the decomposition of soluble organic C in responses to the combined perturbations (Liu & Greaver, 2010).

Defoliation had little impact on soil microbial community, while fertilisation greatly increased bacterial and fungal PLFA biomass, but with little effect on the ratio of fungal to bacterial PLFAs. Moreover, when defoliation and fertilisation were combined, these effects followed the similar trends as fertilisation but with less amplitude. These results indicate that fertilisation rather than defoliation has potential to stimulate soil bacterial and fungal communities without altering soil microbial community structure, as also found by others (Fanin et al., 2015; Li et al., 2020; Liang et al., 2011). However, previous studies have reported that fertiliser application, especially of phosphorus, can decrease the diversity and abundance of arbuscular mycorrhizal fungi, which influences phosphorus transfer from microbes to host plants, as well as long‐term SOM accumulation via melanised hyphae (Frater et al., 2018; Labouyrie et al., 2023; Oelmann et al., 2021; Ryan & Graham, 2018).

Previous studies have reported a negative relationship between total PLFA and defoliation intensity and a decrease of the relative abundance of saprophytic fungal group to total PLFA induced by clipping (Shahzad et al., 2012; Williamson & Wardle, 2007). Nevertheless, most studies have revealed that defoliation does not affect soil microbial community structure (Kotas et al., 2017; Lemanski & Scheu, 2015; Williamson & Wardle, 2007). The minor impact of the combined treatment on microbial community structure, in terms of fungal and bacterial biomass and the relative abundance of fungal to bacterial PLFAs, cannot exclude the possibility that certain microbial taxa could respond to combined perturbations. For example, Sveen et al. (2021) found that the combined effects of herbivory and N‐eutrophication had a stronger effect on soil microbiome such as bacterial phyla than each treatment. Nevertheless, further studies are needed to explore the consequences of combined perturbations for structure of soil microbiome.

### 4.3 Effects of root traits on soil properties under perturbations

Having shown that defoliation and fertilisation impact root traits in contrasting ways, with consequences for soil chemical properties and microbial communities, we tested how these responses contribute to soil aggregation. Root length density was one of most important root traits of increasing soil aggregate stability against fast wetting and slow wetting. This indicates that root length density improves the stability of soil aggregates against the breakdown caused by the compression of entrapped air within aggregates (Gould et al., 2016; ISO, 2012; Le Bissonnais, 1996) and by microcracking (Gould et al., 2016; Hao et al., 2020). In contrast, higher specific root length was found to relate to lower aggregate stability against breakdown caused by microcracking and raindrop impact (Le Bissonnais, 1996). Our results confirm the crucial role of root traits especially root length density and specific root length in regulating soil aggregate stability (Bardgett et al., 2014; Gould et al., 2016).

Our results suggest that the variations in root traits triggered by perturbations result in the changes of their role in regulating soil aggregate stability. For example, root length density played a relatively more important role in increasing soil aggregate stability under perturbation treatments compared with the control. Underlying potential mechanisms for this may relate to the interlink between SOM stabilisation, roots, and root‐associated mycorrhizal fungi (Le Bissonnais et al., 2018; Poirier, Roumet, Angers, et al., 2018; Rillig et al., 2015). Plant roots contribute to soil aggregation through promoting SOM stabilisation by increasing plant C inputs such as root‐derived organic compounds and root litter (Gould et al., 2016; Poirier, Roumet, & Munson, 2018) but also through enmeshing soil particles by collaborating with root‐associated mycorrhizal fungi (Poirier, Roumet, Angers, et al., 2018; Rillig et al., 2015). Particularly, high specific root length indicates “do‐it‐yourself” below‐ground resource acquisition strategies, while increasing root diameter indicates “outsourcing” of resource uptake to mycorrhizal fungi (Bergmann et al., 2020). Mycorrhizal fungi can affect soil aggregate stability by cooperating with plant roots for optimising resource uptake from soil (Bergmann et al., 2020), as well as by their fungal hyphae and polysaccharides (Bardgett et al., 2014; Rillig et al., 2015).

It is well known that plant roots and their fungal symbionts play a vital role in the formation and stabilisation of soil aggregates (Angulo et al., 2022; Demenois et al., 2018; Ossowicki et al., 2021; Rillig et al., 2015; Wang et al., 2020), but our results indicate that their roles in soil aggregation can be modified by perturbations such as defoliation and fertilisation. For example, we found that defoliation inhibited root growth, and potentially root‐associated fungi, as well as removing above‐ground material, which could reduce plant C input to soil aggregates, and reduce SOM stabilisation. In contrast, fertilisation increased both shoot and root biomass, potentially increasing plant C inputs to soil and their incorporation into aggregates. Furthermore, it has been reported that nitrogen addition increases C concentration in macroaggregates, and that C stabilisation in macroaggregates is closely linked to plant biomass, soil available nutrients, and the ratio of fungal to bacterial PLFA biomass (Poirier, Roumet, Angers, et al., 2018; Tisdall & Oades, 1982; Wang et al., 2020). However, C stabilisation in microaggregates is more related to physical protection and restricted fungal mineralisation (Poirier, Roumet, Angers, et al., 2018; Tisdall & Oades, 1982; Wang et al., 2020).

Although individual soil properties such as soil water content had direct or indirect effects on aggregate stability, the total contribution of soil properties to explaining aggregate stability under perturbations was relatively low compared to the role of root traits. Increasing soil water content decreased soil aggregate stability while soil nutrients (i.e. DOC, SOM, plant available nitrogen) and microbial community (i.e. microbial biomass C and the ratio of fungal to bacterial PLFA biomass) had a positive relationship with soil aggregate stability. This resulted in a lower total contribution of soil properties than root traits under defoliation and fertilisation, given the substantial and positive contributions of root length density and root average diameter to soil aggregate stability. Nevertheless, caution is needed in drawing broad conclusions about the relative roles of soil properties and root traits in determining soil aggregate stability, given that while we examined a wide range of plant species, they were grown in a single silt loam soil. Previous studies have emphasised the importance of soil properties such as soil organic C, soil water content and the relative abundance of fungi and bacteria to soil aggregation (Bottinelli et al., 2017; Liao et al., 2020). However, Xiao et al. (2020) found the stronger positive effects of plant roots on soil aggregation than soil in a secondary‐succession grassland with little human interference. Nevertheless, few studies have examined the relative importance of root traits and soil properties to soil aggregate stability and have compared their contributions under perturbations. By disentangling the complex effect pathways of root traits on soil properties and clarify their importance for soil aggregate stability, our results demonstrate that perturbations enhance the contributions of root traits to soil aggregate stability despite the important roles of individual soil properties.

Collectively, our findings from a wide range of grassland plant species with different resource acquisition strategies, indicate that root traits, including root length density and specific root length, consistently respond to defoliation and fertilisation, albeit in opposing ways. We also show that these short‐term root trait responses influence a wide range of biological, chemical and physical soil properties key to ecosystem functioning. Moreover, both defoliation and fertilisation, individually or in combination, enhanced the direct contributions of root traits to soil properties, especially soil aggregate stability, a key physical property of soil. Overall, our findings advance our understanding of how perturbations common to grasslands can modify root traits of a broad range of grassland plant species with consequences for soil abiotic and biotic properties that underpin ecosystem functioning. Our results enforce the notion that root traits act as important determinants of below‐ground properties, but also that perturbations common to grasslands, namely defoliation and fertilisation, strongly modify these responses in contrasting ways.

## AUTHOR CONTRIBUTIONS

The authors declare the following contribution to the paper: Yan Liu: Conceptualisation, methodology, formal analysis, writing—original draft, writing—review & editing, visualisation, funding acquisition. Irene Cordero: Conceptualisation, methodology, writing—review & editing, supervision. Richard D. Bardgett: Conceptualisation, methodology, writing—review & editing, supervision, funding acquisition.

## CONFLICT OF INTEREST STATEMENT

The authors declare that there is no conflict of interest. Richard Bardgett is a Senior Editor of Journal of Ecology, but took no part in the peer review and decision‐making processes for this paper.

### PEER REVIEW

The peer review history for this article is available at https://www.webofscience.com/api/gateway/wos/peer‐review/10.1111/1365‐2745.14215.

## Supporting information


**Table S1.** Variation in root traits over plant species under control conditions.
**Table S2.** Variation in shoot traits over plant species under control conditions.
**Table S3.** Variation in soil physicochemical and biological properties over plant species under control conditions.
**Table S4.** Relative perturbation effects on plant traits over plant growth strategies.

## Data Availability

Data are available in a Figshare repository at https://doi.org/10.6084/m9.figshare.24228364.v2 (Liu, 2023) and https://doi.org/10.6084/m9.figshare.21394965.v2. (Liu, 2022).
