# Supplementary material for: Defoliation and fertilisation differentially moderate root trait effects on soil abiotic and biotic properties
Source: J Ecol. 2023 Oct 25;111(12):2733–49. doi: 10.1111/1365-2745.14215 (PMC10952586; doi:10.1111/1365-2745.14215)
Supplement: Supplementary file 1 — Table S1. Variation in root traits over plant species under control conditions. Table S2. Variation in shoot traits over plant species under control conditions. Table S3. Variation in soil physicochemical and biological properties over plant species under control conditions. Table S4. Relative perturbation effects on plant traits over plant growth strategies. [file JEC-111-2733-s001.docx]

## Supporting Information

Article title: Defoliation and fertilisation differentially moderate root trait effects on soil abiotic and biotic properties

Authors: Yan Liu, Irene Cordero, Richard D. Bardgett

Supporting Information for this article is as follows:

**Table S1** Variation in root traits over plant species under control conditions.

**Table S2** Variation in shoot traits over plant species under control conditions.

**Table S3** Variation in soil physicochemical and biological properties over plant species under control conditions.

**Table S4** Relative perturbation effects on plant traits over plant growth strategies.

**Table S1** Variation in root traits over plant species under control conditions.

| Species | AD | SRL | DMC | RTD | RLD | RMD | VFR | FR | RNC | RCC | RCN | RB |
| --- | --- | --- | --- | --- | --- | --- | --- | --- | --- | --- | --- | --- |
|  | (mm) | (m g^-1^) |  | (g cm^-3^) | (cm cm^-3^) | (g dm^-3^) | (%) | (%) | (%) | (%) |  | (g) |
| Conservative | | | | | | | | | | | | |
| DC | 0.22 ± 0.01 | 137.77 ± 21.07 | 0.2 ± 0.05 | 0.20 ± 0.01 | 28.10 ± 1.72 | 2.06 ± 0.20 | 91.38 ± 1.44 | 7.59 ± 1.22 | 0.48 ± 0.04 | 44.52 ± 0.85 | 94.23 ± 7.26 | 0.85 ± 0.08 |
|  | ab | ab | ab | ab | ns | ab | ab | ab | ab | ab | abc | ab |
| Fr | 0.22 ± 0.01 | 192.58 ± 15.71 | 0.19 ± 0.03 | 0.14 ± 0.00 | 42.72 ± 8.16 | 2.22 ± 0.37 | 93.92 ± 0.92 | 5.24 ± 0.68 | 0.64 ± 0.04 | 45.20 ± 0.52 | 71.13 ± 5.08 | 0.91 ± 0.14 |
|  | ab | a | ab | b | ns | ab | a | b | ab | a | abc | ab |
| Lv | 0.34 ± 0.02 | 79.53 ± 14.63 | 0.17 ± 0.06 | 0.14 ± 0.02 | 14.40 ± 4.32 | 1.81 ± 0.39 | 85.68 ± 2.12 | 12.05 ± 1.2 | 0.88 ± 0.07 | 44.29 ± 0.68 | 50.83 ± 3.44 | 0.74 ± 0.16 |
|  | ab | ab | ab | b | ns | ab | b | a | a | ab | c | ab |
| Pl | 0.28 ± 0.02 | 138.11 ± 25.31 | 0.13 ± 0.02 | 0.12 ± 0.01 | 42.52 ± 8.21 | 3.08 ± 0.18 | 91.57 ± 2.37 | 7.51 ± 1.96 | 0.70 ± 0.03 | 45.35 ± 0.57 | 64.86 ± 2.19 | 1.31 ± 0.07 |
|  | ab | ab | b | b | ns | ab | ab | ab | ab | a | abc | ab |
| Intermediate | | | | | | | | | | | | |
| Cc | 0.26 ± 0.02 | 158.85 ± 20.94 | 0.22 ± 0.04 | 0.12 ± 0.01 | 36.70 ± 12.18 | 2.29 ± 0.54 | 91.92 ± 2.34 | 6.38 ± 1.43 | 0.46 ± 0.04 | 45.15 ± 0.26 | 98.81 ± 9.57 | 0.96 ± 0.22 |
|  | ab | ab | ab | b | ns | ab | ab | ab | ab | a | ab | ab |
| Lp | 0.22 ± 0.02 | 161.35 ± 19.3 | 0.17 ± 0.03 | 0.16 ± 0.02 | 47.00 ± 8.29 | 2.95 ± 0.60 | 91.59 ± 1.49 | 7.24 ± 1.12 | 0.53 ± 0.04 | 44.81 ± 1.15 | 85.38 ± 7.44 | 1.21 ± 0.24 |
|  | ab | ab | ab | b | ns | ab | ab | ab | ab | ab | abc | ab |
| Cn | 0.35 ± 0.03 | 51.51 ± 12.85 | 0.18 ± 0.05 | 0.21 ± 0.02 | 18.07 ± 5.35 | 3.56 ± 0.76 | 88.48 ± 2.89 | 9.36 ± 2.44 | 0.50 ± 0.09 | 43.27 ± 0.35 | 88.57 ± 15.04 | 1.46 ± 0.30 |
|  | a | b | ab | ab | ns | a | ab | ab | ab | ab | abc | a |
| Ra | 0.31 ± 0.02 | 56.97 ± 15.88 | 0.22 ± 0.06 | 0.25 ± 0.04 | 13.53 ± 5.44 | 2.47 ± 1.15 | 92.06 ± 1.31 | 5.17 ± 0.64 | 0.50 ± 0.08 | 42.28 ± 0.48 | 85.08 ± 12.21 | 1.02 ± 0.46 |
|  | ab | ab | ab | ab | ns | ab | ab | b | ab | b | abc | ab |
| Exploitative | | | | | | | | | | | | |
| Ac | 0.22 ± 0.01 | 174.69 ± 18.27 | 0.19 ± 0.02 | 0.15 ± 0.01 | 43.18 ± 6.64 | 2.51 ± 0.54 | 92.03 ± 0.69 | 6.63 ± 0.37 | 0.44 ± 0.07 | 45.44 ± 0.25 | 105.00 ± 15.13 | 1.04 ± 0.21 |
|  | ab | ab | ab | b | ns | ab | ab | ab | b | a | a | ab |
| Pp | 0.21 ± 0.03 | 191.47 ± 44.75 | 0.2 ± 0.06 | 0.15 ± 0.00 | 39.21 ± 4.67 | 2.15 ± 0.67 | 92.97 ± 1.87 | 6.09 ± 1.56 | 0.56 ± 0.09 | 44.32 ± 1.47 | 81.23 ± 14.82 | 0.90 ± 0.32 |
|  | b | ab | ab | b | ns | ab | a | b | ab | ab | abc | ab |
| Lh | 0.27 ± 0.01 | 137.34 ± 15.09 | 0.14 ± 0.06 | 0.13 ± 0.01 | 15.78 ± 4.61 | 1.14 ± 0.24 | 93.31 ± 1.04 | 5.52 ± 0.75 | 0.87 ± 0.08 | 45.22 ± 0.35 | 52.30 ± 4.69 | 0.46 ± 0.10 |
|  | ab | ab | b | b | ns | b | a | b | a | a | bc | b |
| Ruma | 0.24 ± 0.02 | 52.65 ± 14.59 | 0.33 ± 0.08 | 0.46 ± 0.07 | 25.28 ± 4.85 | 5.13 ± 1.72 | 93.18 ± 1.62 | 5.08 ± 0.97 | 0.66 ± 0.14 | 44.58 ± 0.46 | 70.31 ± 16.15 | 2.05 ± 0.65 |
|  | ab | b | a | a | ns | a | a | b | ab | ab | abc | a |

Plant species: Dc, *Deschampsia cespitosa*; Fr, *Festuca rubra*; Lv, *Leucanthemum vulgare*; Pl, *Plantago lanceolata*; Cc, *Cynosurus cristatus*; Lp, *Lolium perenne*; Cn, *Centaurea nigra*; Ra, *Ranunculus acris*; Ac, *Agrostis capillaris*; Pp, *Phleum pratense*; Lh, *Leontodon hispidus*; Ruma, *Rumex acetosa*

Root traits: AD, root average diameter; SRL, specific root length; DMC, root dry matter content; RTD, root tissue density; RLD, root length density; RMD, root mass density; VFR, very fine roots (<0.5 mm); FR, fine roots (0.5-1 mm); RNC, root nitrogen content; RCC, root carbon content; RCN, root carbon: nitrogen ratio; RB, root biomass

Different letters indicate significant differences among plant species at the *P* < 0.05 level by LSD test. Asterisks show significance in one-way ANOVA test (ns: insignificant; **p* < 0.05; ***p* < 0.01; ****p* < 0.001).

**Table S2** Variation in shoot traits over plant species under control conditions.

| Species | AGB | RMF | RSR | LNC | LCC | LCN | SLA |
| --- | --- | --- | --- | --- | --- | --- | --- |
|  | (g) |  |  | (%) | (%) |  | (m^2^ kg^-1^) |
| Conservative | | | | | | | |
| DC | 1.39 ± 0.41 | 0.39 ± 0.07 | 0.65 ± 0.20 | 1.05 ± 0.14 | 44.31 ± 0.24 | 42.61 ± 5.21 | 12.53 ± 1.52 |
|  | ab | ab | ab | ab | a | ab | b |
| Fr | 0.62 ± 0.06 | 0.59 ± 0.04 | 1.47 ± 0.22 | 1.15 ± 0.11 | 43.99 ± 0.24 | 38.69 ± 3.64 | 14.32 ± 1.22 |
|  | ab | ab | ab | ab | a | ab | ab |
| Lv | 0.88 ± 0.46 | 0.48 ± 0.08 | 0.94 ± 0.28 | 0.85 ± 0.11 | 43.03 ± 0.20 | 51.36 ± 7.37 | 13.95 ± 1.19 |
|  | ab | ab | ab | ab | ab | ab | ab |
| Pl | 1.40 ± 0.44 | 0.49 ± 0.10 | 1.05 ± 0.49 | 0.69 ± 0.06 | 42.66 ± 0.83 | 62.65 ± 5.68 | 13.20 ± 2.12 |
|  | ab | ab | ab | b | ab | a | b |
| Intermediate | | | | | | | |
| Cc | 0.92 ± 0.06 | 0.50 ± 0.05 | 1.03 ± 0.20 | 1.35 ± 0.23 | 42.24 ± 0.41 | 31.97 ± 5.46 | 24.32 ± 2.12 |
|  | ab | ab | ab | ab | ab | ab | ab |
| Lp | 1.09 ± 0.57 | 0.54 ± 0.14 | 1.41 ± 0.97 | 1.37 ± 0.27 | 42.93 ± 0.27 | 32.19 ± 6.18 | 20.54 ± 5.2 |
|  | ab | ab | ab | a | ab | ab | ab |
| Cn | 0.70 ± 0.20 | 0.68 ± 0.02 | 2.11 ± 0.24 | 1.17 ± 0.16 | 43.17 ± 0.31 | 37.64 ± 5.53 | 15.84 ± 1.28 |
|  | ab | a | ab | ab | ab | ab | ab |
| Ra | 0.44 ± 0.10 | 0.68 ± 0.06 | 2.22 ± 0.56 | 1.30 ± 0.31 | 42.69 ± 0.53 | 34.07 ± 7.71 | 22.64 ± 2.34 |
|  | b | a | ab | ab | ab | ab | ab |
| Exploitative | | | | | | | |
| Ac | 1.93 ± 0.76 | 0.36 ± 0.06 | 0.57 ± 0.13 | 1.12 ± 0.16 | 44.11 ± 0.31 | 40.01 ± 4.97 | 37.11 ± 30.12 |
|  | a | ab | b | ab | a | ab | a |
| Pp | 1.96 ± 0.37 | 0.31 ± 0.08 | 0.47 ± 0.17 | 0.84 ± 0.08 | 43.83 ± 0.28 | 52.36 ± 5.01 | 17.44 ± 6.32 |
|  | a | b | b | ab | a | a | ab |
| Lh | 0.60 ± 0.18 | 0.43 ± 0.06 | 0.78 ± 0.19 | 1.6 ± 0.20 | 40.9 ± 0.44 | 25.81 ± 2.73 | 25.77 ± 3.83 |
|  | ab | ab | ab | a | b | b | ab |
| Ruma | 0.74 ± 0.20 | 0.73 ± 0.04 | 2.81 ± 0.51 | 1.44 ± 0.49 | 43.46 ± 1.24 | 32.79 ± 10 | 24.45 ± 7.94 |
|  | ab | a | a | a | a | ab | ab |

Plant species: Dc, *Deschampsia cespitosa*; Fr, *Festuca rubra*; Lv, *Leucanthemum vulgare*; Pl, *Plantago lanceolata*; Cc, *Cynosurus cristatus*; Lp, *Lolium perenne*; Cn, *Centaurea nigra*; Ra, *Ranunculus acris*; Ac, *Agrostis capillaris*; Pp, *Phleum pratense*; Lh, *Leontodon hispidus*; Ruma, *Rumex acetosa*

Shoot traits: AGB, above-ground biomass; RMF, root mass fraction; RSR, root: shoot ratio; LNC, leaf nitrogen content; LCC, leaf carbon content; LCN, leaf carbon: nitrogen ratio; SLA, specific leaf area

Different letters indicate significant differences among plant species at the *P* < 0.05 level by LSD test. Asterisks show significance in one-way ANOVA test (ns: insignificant; **p* < 0.05; ***p* < 0.01; ****p* < 0.001).

**Table S3** Variation in soil physicochemical and biological properties over plant species under control conditions.

| Species | MWD1 | MWD2 | MWD3 | SWC | DOC | TC | TN | SOM | NH_4_ | NO_3_ | MBC | F | B | F/B ratio |
| --- | --- | --- | --- | --- | --- | --- | --- | --- | --- | --- | --- | --- | --- | --- |
|  | (mm) | (mm) | (mm) |  | (mg kg^-1^) | (%) | (%) | (%) | (mg kg^-1^) | (mg kg^-1^) | (mg kg^-1^) | (nmol g^-1^) | (nmol g^-1^) |  |
| Conservative | | | | | | | | | | | | | | |
| DC | 2.77 ± 0.17 | 3.41 ± 0.05 | 3.19 ± 0.12 | 0.50 ± 0.03 | 28.65 ± 4.07 | 3.26 ± 0.37 | 0.27 ± 0.03 | 7.92 ± 0.24 | 4.23 ± 0.19 | 3.27 ± 0.54 | 190.55 ± 83.41 | 6.54 ± 2.12 | 197.77 ± 22.06 | 0.03 ± 0.01 |
|  | ns | ns | ns | ns | ns | ns | ns | ns | ns | ns | ns | ns | ns | a |
| Fr | 2.92 ± 0.19 | 3.40 ± 0.12 | 3.28 ± 0.16 | 0.46 ± 0.02 | 27.04 ± 5.95 | 3.81 ± 0.53 | 0.32 ± 0.04 | 8.25 ± 0.32 | 3.52 ± 0.90 | 2.11 ± 1.69 | 214.01 ± 60.30 | 8.91 ± 4.05 | 235.96 ± 107.45 | 0.04 ± 0.00 |
|  | ns | ns | ns | ns | ns | ns | ns | ns | ns | ns | ns | ns | ns | ab |
| Lv | 2.91 ± 0.25 | 3.43 ± 0.03 | 3.21 ± 0.06 | 0.50 ± 0.03 | 23.23 ± 3.97 | 3.56 ± 0.38 | 0.30 ± 0.03 | 8.11 ± 0.44 | 3.72 ± 0.54 | 0.67 ± 0.12 | 176.32 ± 34.06 | 13.28 ± 3.82 | 219.88 ± 94.79 | 0.06 ± 0.01 |
|  | ns | ns | ns | ns | ns | ns | ns | ns | ns | ns | ns | ns | ns | ab |
| Pl | 3.10 ± 0.08 | 3.38 ± 0.12 | 3.24 ± 0.08 | 0.45 ± 0.04 | 27.94 ± 4.66 | 3.58 ± 0.31 | 0.29 ± 0.03 | 8.06 ± 0.51 | 4.55 ± 0.34 | 0.57 ± 0.13 | 230.44 ± 39.39 | 15.21 ± 7.38 | 200.88 ± 70.60 | 0.07 ± 0.01 |
|  | ns | ns | ns | ns | ns | ns | ns | ns | ns | ns | ns | ns | ns | ab |
| Intermediate | | | | | | | | | | | | | | |
| Cc | 2.86 ± 0.26 | 3.41 ± 0.04 | 3.23 ± 0.11 | 0.52 ± 0.04 | 30.91 ± 2.68 | 2.94 ± 0.60 | 0.23 ± 0.06 | 8.19 ± 0.37 | 3.65 ± 0.50 | 1.25 ± 1.21 | 200.54 ± 14.11 | 14.64 ± 6.41 | 253.90 ± 91.45 | 0.06 ± 0.02 |
|  | ns | ns | ns | ns | ns | ns | ns | ns | ns | ns | ns | ns | ns | ab |
| Lp | 3.16 ± 0.12 | 3.44 ± 0.02 | 3.34 ± 0.06 | 0.50 ± 0.03 | 31.91 ± 7.22 | 3.59 ± 1.26 | 0.29 ± 0.11 | 8.12 ± 0.31 | 4.10 ± 0.37 | 1.51 ± 0.36 | 190.30 ± 36.71 | 12.29 ± 7.48 | 190.15 ± 69.44 | 0.06 ± 0.02 |
|  | ns | ns | ns | ns | ns | ns | ns | ns | ns | ns | ns | ns | ns | ab |
| Cn | 2.67 ± 0.37 | 3.40 ± 0.05 | 3.25 ± 0.10 | 0.46 ± 0.05 | 19.79 ± 1.64 | 3.65 ± 0.38 | 0.30 ± 0.04 | 7.90 ± 0.30 | 3.59 ± 1.09 | 0.60 ± 0.08 | 241.49 ± 8.49 | 9.51 ± 4.18 | 194.14 ± 57.05 | 0.05 ± 0.01 |
|  | ns | ns | ns | ns | ns | ns | ns | ns | ns | ns | ns | ns | ns | ab |
| Ra | 3.02 ± 0.07 | 3.39 ± 0.03 | 3.29 ± 0.05 | 0.46 ± 0.05 | 23.23 ± 1.49 | 3.92 ± 1.06 | 0.33 ± 0.09 | 8.13 ± 0.57 | 4.24 ± 0.76 | 0.66 ± 0.14 | 228.77 ± 72.54 | 10.54 ± 5.44 | 208.59 ± 90.73 | 0.05 ± 0.01 |
|  | ns | ns | ns | ns | ns | ns | ns | ns | ns | ns | ns | ns | ns | ab |
| Exploitative | | | | | | | | | | | | | | |
| Ac | 2.76 ± 0.29 | 3.34 ± 0.07 | 3.16 ± 0.10 | 0.48 ± 0.03 | 28.84 ± 6.75 | 3.17 ± 0.33 | 0.26 ± 0.02 | 8.11 ± 0.29 | 3.80 ± 1.38 | 0.66 ± 0.08 | 253.19 ± 6.26 | 14.03 ± 1.64 | 241.95 ± 53.71 | 0.06 ± 0.01 |
|  | ns | ns | ns | ns | ns | ns | ns | ns | ns | ns | ns | ns | ns | ab |
| Pp | 2.64 ± 0.19 | 3.40 ± 0.03 | 3.23 ± 0.09 | 0.51 ± 0.02 | 27.55 ± 4.12 | 3.17 ± 0.23 | 0.27 ± 0.02 | 7.98 ± 0.27 | 4.20 ± 1.27 | 1.79 ± 0.73 | 219.16 ± 17.21 | 8.88 ± 3.78 | 226.54 ± 61.13 | 0.04 ± 0.01 |
|  | ms | ns | ns | ns | ns | ns | ns | ns | ns | ns | ns | ns | ns | ab |
| Lh | 2.79 ± 0.43 | 3.38 ± 0.07 | 3.23 ± 0.05 | 0.51 ± 0.02 | 21.97 ± 1.36 | 3.26 ± 0.55 | 0.27 ± 0.05 | 7.81 ± 0.16 | 3.93 ± 1.14 | 0.75 ± 0.14 | 222.95 ± 56.82 | 15.85 ± 4.61 | 237.01 ± 25.98 | 0.07 ± 0.02 |
|  | ns | ns | ns | ns | ns | ns | ns | ns | ns | ns | ns | ns | ns | ab |
| Ruma | 2.76 ± 0.11 | 3.36 ± 0.07 | 3.23 ± 0.07 | 0.50 ± 0.02 | 22.20 ± 4.25 | 3.88 ± 0.21 | 0.32 ± 0.01 | 7.98 ± 0.24 | 3.15 ± 1.48 | 2.82 ± 2.00 | 189.85 ± 21.37 | 15.36 ± 5.62 | 219.12 ± 31.23 | 0.07 ± 0.03 |
|  | ns | ns | ns | ns | ns | ns | ns | ns | ns | ns | ns | ns | ns | b |

Plant species: Dc, *Deschampsia cespitosa*; Fr, *Festuca rubra*; Lv, *Leucanthemum vulgare*; Pl, *Plantago lanceolata*; Cc, *Cynosurus cristatus*; Lp, *Lolium perenne*; Cn, *Centaurea nigra*; Ra, *Ranunculus acris*; Ac, *Agrostis capillaris*; Pp, *Phleum pratense*; Lh, *Leontodon hispidus*; Ruma, *Rumex acetosa*

MWD1, mean weight diameter (slaking); MWD2, mean weight diameter (microcracking); MWD3, mean weight diameter (mechanical breakdown); SWC, gravimetric soil water content; DOC, dissolved organic carbon; MBC, soil microbial biomass carbon; TC, total soil carbon content; TN, total soil nitrogen content; SOM, soil organic matter; NH_4_, plant available ammonia; NO_3_, plant available nitrate; F, fungal PLFA biomass; B, bacterial PLFA biomass; F/B ratio, the ratio of fungal to bacterial PLFA biomass.

Different letters indicate significant differences among plant species at the *P* < 0.05 level by LSD test. Asterisks show significance in one-way ANOVA test (ns: insignificant; **p* < 0.05; ***p* < 0.01; ****p* < 0.001).

**Table S4** Relative perturbation effects on plant traits over plant growth strategies.

|  | Relative defoliation effect | | | |  | Relative fertilisation effect | | | |  | Relative combined effect | | | |
| --- | --- | --- | --- | --- | --- | --- | --- | --- | --- | --- | --- | --- | --- | --- |
|  | Conservative | Intermediate | Exploitative | P-value |  | Conservative | Intermediate | Exploitative | P-value |  | Conservative | Intermediate | Exploitative | P-value |
| AD | 0.02 ± 0.28 | 0.33 ± 0.37 | -0.08 ± 0.08 | 0.551 |  | -0.4 ± 0.31 | -0.68 ± 0.31 | -0.7 ± 0.38 | 0.148 |  | 0.48 ± 0.96 | 0.55 ± 0.53 | 0 ± 0.05 | **0.024 *** |
| SRL | 0.39 ± 0.15 | 0.39 ± 0.33 | 0.45 ± 0.43 | 0.648 |  | -0.18 ± 0.14 | -0.2 ± 0.17 | -0.19 ± 0.18 | 0.887 |  | 0.19 ± 0.12 | 0.18 ± 0.5 | 0.33 ± 0.35 | 0.299 |
| DMC | -0.11 ± 0.16 | -0.22 ± 0.2 | -0.04 ± 0.27 | 0.414 |  | -0.03 ± 0.1 | -0.03 ± 0.12 | -0.05 ± 0.14 | 0.615 |  | -0.14 ± 0.11 | -0.16 ± 0.29 | -0.03 ± 0.36 | 0.260 |
| RTD | -0.07 ± 0.11 | -0.22 ± 0.23 | -0.13 ± 0.24 | 0.389 |  | 0.05 ± 0.06 | -0.04 ± 0.09 | 0.04 ± 0.19 | 0.785 |  | -0.17 ± 0.09 | -0.3 ± 0.23 | -0.21 ± 0.19 | 0.592 |
| RLD | -0.36 ± 0.14 | -0.45 ± 0.2 | -0.29 ± 0.13 | 0.374 |  | 0.71 ± 0.49 | 0.67 ± 0.33 | 0.85 ± 0.27 | 0.404 |  | -0.2 ± 0.22 | -0.29 ± 0.21 | -0.12 ± 0.14 | 0.334 |
| RMD | -0.54 ± 0.08 | -0.57 ± 0.23 | -0.5 ± 0.16 | 0.594 |  | 1.15 ± 0.66 | 1.16 ± 0.51 | 1.39 ± 0.7 | 0.362 |  | -0.33 ± 0.15 | -0.3 ± 0.39 | -0.34 ± 0.08 | 0.922 |
| VFR | -0.11 ± 0.15 | -0.04 ± 0.05 | -0.11 ± 0.13 | 0.981 |  | -0.21 ± 0.13 | -0.19 ± 0.06 | -0.21 ± 0.06 | 0.935 |  | -0.14 ± 0.12 | -0.13 ± 0.13 | -0.09 ± 0.22 | 0.384 |
| FR | -0.28 ± 0.12 | 0.08 ± 0.34 | -0.21 ± 0.33 | 0.644 |  | -0.03 ± 0.26 | 0.12 ± 0.27 | -0.02 ± 0.09 | 0.953 |  | 0 ± 0.27 | 0.14 ± 0.1 | -0.01 ± 0.32 | 0.957 |
| RNC | 0.18 ± 0.11 | 0.4 ± 0.46 | 0.1 ± 0.06 | 0.417 |  | 0.1 ± 0.1 | 0.33 ± 0.25 | 0.24 ± 0.22 | 0.157 |  | 0.47 ± 0.06 | 0.89 ± 0.81 | 0.43 ± 0.29 | 0.836 |
| RCC | 0 ± 0.01 | 0.01 ± 0.02 | -0.01 ± 0 | 0.273 |  | -0.02 ± 0.01 | 0 ± 0.01 | -0.02 ± 0.03 | 0.874 |  | 0 ± 0 | 0.01 ± 0.02 | 0.01 ± 0.01 | 0.143 |
| RCN | -0.15 ± 0.08 | -0.22 ± 0.25 | -0.1 ± 0.05 | 0.452 |  | -0.07 ± 0.08 | -0.22 ± 0.13 | -0.2 ± 0.13 | 0.052 |  | -0.32 ± 0.02 | -0.39 ± 0.25 | -0.29 ± 0.14 | 0.609 |
| RMF | -0.36 ± 0.11 | -0.34 ± 0.17 | -0.22 ± 0.07 | **0.013 *** |  | -0.3 ± 0.17 | -0.22 ± 0.11 | -0.15 ± 0.25 | **0.039 *** |  | -0.6 ± 0.1 | -0.56 ± 0.17 | -0.5 ± 0.17 | 0.063 |
| AGB | -0.03 ± 0.24 | -0.08 ± 0.09 | -0.14 ± 0.33 | 0.361 |  | 2.73 ± 0.54 | 2.89 ± 0.89 | 2.45 ± 0.96 | 0.393 |  | 1.56 ± 0.53 | 1.85 ± 0.42 | 1.25 ± 1.04 | 0.251 |
| RB | -0.54 ± 0.08 | -0.57 ± 0.23 | -0.47 ± 0.14 | 0.32 |  | 1.16 ± 0.7 | 1.19 ± 0.52 | 1.39 ± 0.7 | 0.397 |  | -0.34 ± 0.15 | -0.3 ± 0.39 | -0.34 ± 0.07 | 0.975 |
| RSR | -0.53 ± 0.14 | -0.57 ± 0.2 | -0.37 ± 0.15 | **0.023 *** |  | -0.45 ± 0.2 | -0.43 ± 0.21 | -0.25 ± 0.37 | 0.052 |  | -0.74 ± 0.11 | -0.76 ± 0.15 | -0.65 ± 0.19 | 0.086 |
| LNC | 1.14 ± 0.23 | 0.9 ± 0.31 | 1.29 ± 0.55 | 0.333 |  | 0.77 ± 0.22 | 0.46 ± 0.15 | 0.38 ± 0.13 | **< 0.001** |  | 2.61 ± 0.55 | 1.85 ± 0.62 | 2.34 ± 1.02 | 0.365 |
| LCC | 0.01 ± 0.02 | 0 ± 0 | -0.01 ± 0.02 | **0.013 *** |  | 0.02 ± 0 | 0.01 ± 0.01 | 0.01 ± 0.01 | 0.544 |  | 0.01 ± 0.01 | 0.02 ± 0.01 | 0.02 ± 0.01 | 0.130 |
| LCN | -0.52 ± 0.05 | -0.47 ± 0.09 | -0.55 ± 0.13 | 0.345 |  | -0.42 ± 0.07 | -0.31 ± 0.07 | -0.27 ± 0.08 | **< 0.001** |  | -0.71 ± 0.04 | -0.64 ± 0.07 | -0.68 ± 0.07 | 0.215 |
| SLA | 0.89 ± 0.4 | 0.53 ± 0.19 | 0.78 ± 0.84 | 0.618 |  | 0.24 ± 0.21 | 0.08 ± 0.19 | 0.09 ± 0.3 | 0.124 |  | 1.15 ± 0.26 | 0.62 ± 0.3 | 0.81 ± 0.88 | 0.099 |

Root traits: AD, root average diameter; SRL, specific root length; DMC, root dry matter content; RTD, root tissue density; RLD, root length density; RMD, root mass density; VFR, very fine roots (<0.5mm); FR, fine roots (0.5<<1mm); RNC, root nitrogen content; RCC, root carbon content; RCN, root carbon: nitrogen ratio; RB, root biomass

Other plant traits: AGB, above-ground biomass; RMF, root mass fraction; RSR, root: shoot ratio; LNC, leaf nitrogen content; LCC, leaf carbon content; LCN, leaf carbon: nitrogen ratio; SLA, specific leaf area

Asterisks show significance in one-way ANOVA test (ns: insignificant; **p* < 0.05; ***p* < 0.01; ****p* < 0.001)
